# Flavonoids affect the endophytic bacterial community in *Ginkgo biloba* leaves with increasing altitude

**DOI:** 10.3389/fpls.2022.982771

**Published:** 2022-08-11

**Authors:** Shaodong Fu, Yan Deng, Kai Zou, Shuangfei Zhang, Xueduan Liu, Yili Liang

**Affiliations:** ^1^School of Resource Processing and Bioengineering, Central South University, Changsha, China; ^2^Key Laboratory of Biometallurgy, Ministry of Education, Central South University, Changsha, China; ^3^College of Advanced Materials Engineering, Jiaxing Nanhu University, Jiaxing, China

**Keywords:** *Ginkgo biloba*, flavonoids, endophyte community, network, function genes

## Abstract

Altitude affects plant growth and metabolism, but the effect of altitude on plant endophytic microorganisms is still unclear. In this study, we selected 16 *Ginkgo biloba* trees to study the response of leaves’ endophytes to flavonoids and altitude (from 530 m to 1,310 m). HPLC results showed that flavonoids in *Ginkgo biloba* leaves increased by more than 150% with attitude rising from 530 m to 1,310 m, which revealed a positive correlation with altitude. *Ginkgo biloba* might regulate the increased flavonoids in leaves to resist the increasing light intensity. 16S rDNA sequencing results showed that the endophytic bacterial communities of *Ginkgo biloba* at different altitudes significantly differed. *Ginkgo* leaf endophytes’ alpha diversity decreased with increasing flavonoids content and altitude. The increased flavonoids might increase the environmental pressure on endophytes and affect the endophytic community in *Ginkgo biloba* leaves. The bacterial network in *Ginkgo biloba* leaves became more complex with increasing altitude, which might be one of the strategies of leaf endophytes to cope with increasing flavonoids. Metagenomes results predicted with PICRUSt showed that the abundance of flavonoid biosynthesis and photosynthesis genes were significantly decreased with the increase of flavonoid contents. High flavonoid content in leaves appeared to inhibit microbial flavonoid synthesis. Our findings indicate that altitude can modulate microbial community structure through regulating plant metabolites, which is important to uncovering the interaction of microbes, host and the environment.

## Introduction

Altitude can affect plant growth and metabolism. Previous research showed that flavonoids biosynthesis in ginkgo leaves is affected by altitude (Zou et al., 2019). Endophytes play important roles in plant health and productivity (Leach et al., 2017). Endophytic bacteria spend part or all of their life inside the plant tissues without causing hosts diseases, and they always play positive roles in plant growth (Schulz and Boyle, 2006; White and Torres, 2010). Generally, endophytes are considered as a complement to the plant gene library, helping the hosts adapt to the environment (Vandenkoornhuyse et al., 2015), such as enhancing hosts’ stress tolerance (e.g., drought and salinity) (Malinowski et al., 2008), improving hosts’ resistance to diseases (Clarke et al., 2006), helping hosts with mineral uptake (Malinowski and Alloush GABelesky, 2000) and promoting hosts growth (Schardl et al., 2004). Besides, endophytes can produce the same bioactive metabolites as the host, like alkaloids, steroids, terpenoids and flavonoids (Tan and Zou, 2010; Deshmukh and Verekar, 2012). Host plants procure an apparent modulation immunity by enriching beneficial microbes (Berendsen et al., 2012). It was generally acknowledged that temperature variation mostly affected the endophytic community formation with the elevation increase (Ju et al., 2006). Thus, deciphering the interaction between plants and endophytes attract much attention in ecology and plant sciences.

The regulation of endophytes by plants is an important part of the relationships between plants and microorganisms. Many factors can affect the community of endophytes in the plant, such as temperature (Ju et al., 2006), precipitation (Brosi et al., 2011) and soil property (Lehtonen et al., 2005). But plant secondary metabolites may have the most immediate effect because of the direct contact with endophyte. Benzoxazinoids, defensive secondary metabolites released by cereals’ roots, were reported to alter root-associated fungal and bacterial communities (Saunders and Kohn, 2009; Hu et al., 2018). Sesquiterpenes induced hyphal branching in an arbuscular mycorrhizal fungus (Akiyama et al., 2005). Flavonoids are also essential secondary metabolites in improving plant-microbe interactions (Hassan and Mathesius, 2012; Chin et al., 2018). Previous studies showed that flavonoids could regulate the endophytic microbial community in roots as allelochemicals, nod gene inducers and phytoalexins (Cooper, 2004; Shaw et al., 2006).

Flavonoids are distinguished antioxidants and plant protectants owing to numerous phenolic hydroxyl radicals (Pietta, 2000). They can inhibit the growth of many microorganisms, including *Staphylococcus aureus*, *Escherichia coli*, and many other bacteria (Parvez et al., 2004; Farhadi et al., 2018). Generally, flavonoids (e.g., catechins) show more significant stress on Gram-positive bacteria than Gram-negative bacteria because of different cytoderm structures (Ikigai et al., 1993; Fathima et al., 2016). *Ginkgo* leaves are the main source of flavonoids, so factors that influence the flavonoid content in *Ginkgo* leaves have been investigated extensively. Previous studies revealed that lower temperature promoted the accumulation of flavonoids, but CO_2_ and O_3_ were important inhibitors of foliar flavonoids in *Ginkgo biloba* (Huang et al., 2010; Wang et al., 2014). Moreover, studies found that UV-B promoted flavonoid synthesis in *Ginkgo biloba* leaves (Zhao et al., 2020). Our recent study observed that the elevation and tree age affect flavonoid biosynthesis, and they attribute it to the light enhancement and gene variations (Zou et al., 2019).

Although flavonoids’ influence on microorganisms has gotten much attention, the relationship between flavonoids and endophyte communities in plant leaves is still scarce. In this study, we collected *Ginkgo* leaves with different concentrations of flavonoids at different altitudes. Combining with plant metabolomics and endophyte genomics analysis, we assessed the response of the endophytic community to changes in flavonoids in *Ginkgo* leaves. Our aims were to (i) explore the reasons for the change of flavones with altitude; (ii) analyze the effect of flavonoids on the endophytic community; and (iii) elucidate the mechanism by which flavonoids affect the community.

## Materials and methods

### Sample collection

We selected 16 *Ginkgo biloba* trees in the Hupingshan Nature Reserve, which were rarely affected by human activities, as the research object (Table 1). Ginkgo’s leaves were chosen as our object owing to their abundant flavonoids. The average annual temperature in the nature reserve is 9.2°C. The extreme maximum temperature in the sample sites is 38.2°C. The extreme minimum temperature is −15°C. The average annual sunshine in the sample sites is 1,509.9 h, and the annual precipitation is 1,898.5 mm. All trees grew in the same general area and experienced similar growing conditions. The elevation and the diameter at breast height (DBH) were determined by the Global Positioning System (GPS) instrument and a tape measure. All trees were healthy, alone, and had no shade. The leaves of the ginkgo canopy are divided into two parts longitudinally with the trunk as the axis. The sampling height is 5-8 m. The two parts are evenly sampled. Multiple leaves are mixed into one sample. About 200 g of healthy and mature leaves were collected as samples in each part. Leaves in different parts were considered as different samples. All samples were collected aseptically wearing bioclean gloves on a sunny day with the same weather condition. All samples were divided into two parts. One was frozen in liquid nitrogen immediately and then stored at −80°C for metabolome detection and flavonoid extraction. Another was surface disinfected promptly for the microbiological analysis. Leaves were sterilized with 75% ethanol for 5 min, then washed with sterile water, soaked in 8% NaClO for 2 min and washed with sterile water again (Sahu et al., 2022). After surface sterilizing, dried the leaves surface with sterile filter paper and sterile air on a sterile workbench. All leaves were triturated in liquid nitrogen and stored at −80°C until DNA extraction.

**TABLE 1:** Altitude and other information of sampling trees.

Sample	DBH(m)	Latitude	Longitude	Altitude(m)
S1_1	3.00	N 29°57′22″	E 110°48’40″	530
S1_2	4.00	N 29°57′22″	E 110°48′40″	530
S1_3	3.24	N 29°51′59″	E 110°44′59″	560
S1_4	2.18	N 29°51′59″	E 110°44′59″	560
S2_1	2.00	N 29°56′55″	E 110°39′16″	800
S2_2	2.15	N 29°54′12″	E 110°44′33″	820
S2_3	3.10	N 29°54′12″	E 110°44′33″	820
S2_4	3.18	N 29°53′7″	E 110°42′28″	840
S3_1	2.26	N 29°56′7″	E 110°38′28″	1,000
S3_2	1.92	N 29°55′50″	E 110°37′6″	1,020
S3_3	2.59	N 29°52′7″	E 110°37′28″	1,040
S3_4	4.12	N 26°55′13″	E 110°36′16″	1,040
S4_1	2.40	N 30°5′47″	E 110°48′50″	1,260
S4_2	3.00	N 30°6′46″	E 110°46′29″	1,300
S4_3	3.74	N 30°6′7″	E 110°49′2″	1,300
S4_4	2.65	N 30°9′23″	E 110°44′19″	1,310

### High-performance liquid chromatography

Frozen leaves were dried in an oven at 60°C until the weight was constant (Zhou et al., 1999). Afterward, these leaves were ground into powder smaller than 40 mesh and extracted in a Soxhlet extractor based on Chinese pharmacopeia (2015). 1.000 g dried powder was homogenized in 100 mL chloroform followed by two h reflux at 80°C. The residue was resolved in 50 mL methanol followed by two-hour reflux at 90°C. Then, 20 mL hydrochloride was added to the supernatant, refluxing for 30 min at 90°C and evaporating to dryness. The residue was dissolved in methanol and filtrated through a 0.22 μm membrane for HPLC (Daigle and Conkerton, 1983).

Chromatographic separation was implemented on a Shimadzu LC-20AD Series HPLC system (Shimadzu, Duisburg, Germany) equipped with SIL-20A autosampler, SPD-20A UV-VIS detector. ACQUITY UPLC™ BEH C18 column (217 mm × 2.1 mm, 1.7 μm, Waters, Milford, United States) was connected to the whole detection at 25°C. The mobile phase consisted of 10% 0.05 M sodium acetate (A) and 90% acetonitrile (B). A flow rate of 1.0 mL/min was used. Standard curves were established by a series concentration of the corresponding standard (ChemFaces, Wuhan, China) at 360 nm (Mattila et al., 2000). And the content of total flavonoids was calculated by the formula (Hasler et al., 1992):


$$\begin{array}{c} \mathrm{Total}\mathrm{flavonoids}\\ \;=2.51quercetin+2.64kaempferol+2.39isorhamnetin \end{array}$$

The data were provided in the (Supplementary Table 1).

### Deoxyribonucleic acid extraction, sequencing and sequence analysis

The leaf fragments were suspended in TE buffer and homogenized in a sterilized mortar and pestle with liquid nitrogen (Zhang et al., 2016). DNA was extracted from the homogenized leaf material using the DNeasyR Plant Mini Kit (Product of Germany). To get rid of the distractions of DNA of *Ginkgo* leaves, the DNA was polymerase chain reaction (PCR) amplified using a primer pair 799F (5′-AACMGGATTAGATAC CCKG-3′) and 1115R (5′-AGGGTTGCGCTCGTTG-3′) (Leff et al., 2015). The DNA was cleaned by DNA Clean-Up Kit (OMEGA bio-teak, Norcross, Georgia, United States). High-throughput sequencing of the PCR products was conducted on the Illumina Miseq platform (Miseq PE250).

By comparing the NCBI and Greengene databases, we removed the sequence of the chloroplasts. Raw 16S rRNA gene sequences were processed using the Galaxy | Denglab^1^. These steps included: quality filtering, RDP clustering, sequence alignments and community dissimilarities analysis (Wu et al., 2018). Taxonomic assignment of 16S representative sequences was executed with the RDP (Ribosomal Database Project) classifier according to the Greengene database (Desantis et al., 2006). Resampled 16S OTU subsets (1000 sequences per sample) were to compute alpha diversity and beta diversity. The data presented in the study are deposited in the NCBI repository, accession number PRJNA852885.

### Data analysis

Alpha-diversity indexes, including Shannon index, Simpson evenness, and Pielou evenness, were calculated at Institute for Environmental Genomics (IEG)^2^. Non-metric multidimensional scaling (NMDS) was calculated to assess the beta diversity. The dissimilarity test identified the difference in bacterial communities. Correlation between bacterial community and flavonoids based on the Mantel test, correlation test, and redundancy analysis (RDA) were completed using the Wekemo Bioincloud^3^. OTUs with an average abundance of more than 0.05% was chosen to construct correlation networks by calculating Spearman’s rank correlations with Spearman’s correlation coefficient (*r* > 0.6, *P* < 0.01) (Steinhauser et al., 2008). We used Gephi to visualize the correlation networks (Bastian et al., 2009).

PICRUSt was used as a bioinformatics tool to predict microflora’s abundance of function genes (Langille et al., 2013). In this study, PICRUSt was employed to predict the functions genes of each sample based on 16S rRNA sequencing data. Comparing the sequence data of the 16S Greengene database (Greengenes 13.5), we predicted the community functional gene relative abundance of endophytic microorganisms with reference to the KEGG database.

## Results

### The content of flavonoids in leaves

**FIGURE 1:**
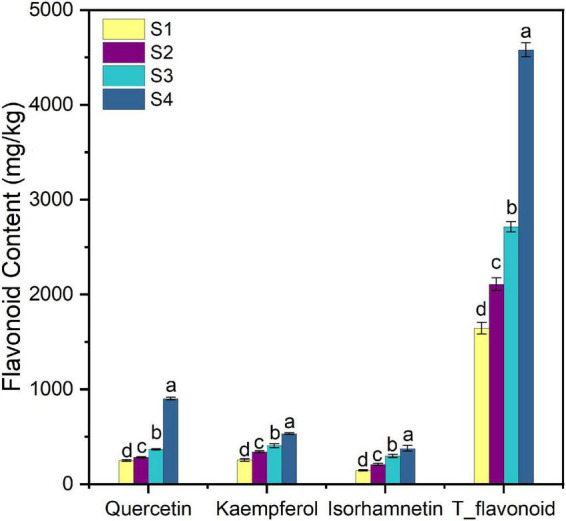
The concentration of three flavonols and total flavonoids in *Ginkgo* leaves at different altitudes. The significant difference among the groups is represented by different letters (*p* < 0.05).

HPLC results showed that quercetin, kaempferol and isorhamnetin were the primary flavonoid metabolites of *Ginkgo* leaves. According to the regression equation of the standard curve (Supplementary Table 2), the content of total flavonoids was calculated (Figure 1). The results showed that the total flavonoids increased by 178.38%, from 1,645.36 mg/kg at S1 to 4580.43 mg/kg at S4 (*p* < 0.05). The three flavonols quercetin, kaempferol and isorhamnetin increase steadily with elevation. The content of quercetin, kaempferol and isorhamnetin in leaves increased by 2.58, 1.11, and 1.71 times from S1 to S4, respectively. The results suggested that Ginkgo flavonoids’ content was increased along the altitude gradient.

### Endophytic community composition and diversity in ginkgo leaves

**FIGURE 2:**
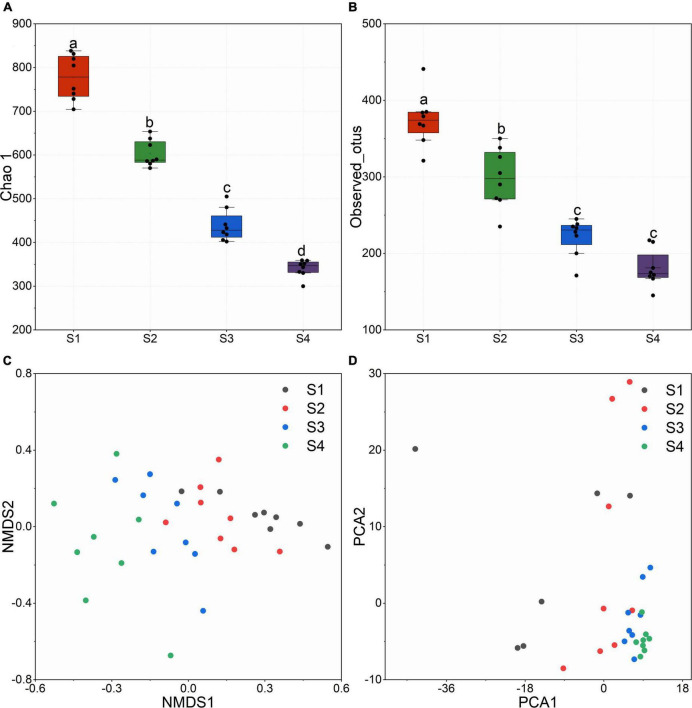
**(A)** Chao 1 index calculated with the OTU table. **(B)** Observed_otus in different sites. **(C)** Non-metric multidimensional scaling (NMDS) based on bray_curtis distance. **(D)** Principal Components Analysis (PCA).

After quality filtering, RDP clustering, sequence alignments and other measures, we obtained 312123 high-quality paired 16S rRNA sequences. At the level of 97% similarity, all sequences were assigned to 3,038 OTUs based on the 16S Greengene database. OTU table was provided in the Supplementary Document. Alpha diversity represented by the Shannon index (Figure 2A) and observed_otus (Figure 2B) revealed that alpha diversity decreased significantly with the increase of flavonoids and the elevation. Samples at lower altitudes had higher species richness and diversity. NMDS and PCA results indicated that the bacterial communities in leaves were dynamic. Samples from the same site huddled together, and samples from different altitudes showed a great distance (Figures 2C,D).

**FIGURE 3:**
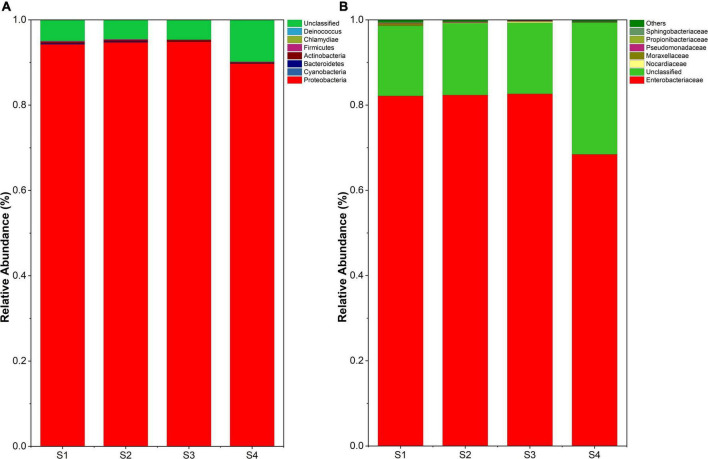
**(A)** Endophyte composition in all samples at the phylum level. **(B)** Endophyte community composition at the family level.

All reads were affiliated with seven bacterial phyla except 5%∼10% sequences unclassified. The dominant phylum was Proteobacteria, accounting for 90%∼95% of the reads (Figure 3A). The samples at lower altitudes had fewer unclassified reads at the phylum level. Bacteria of Actinobacteria and Firmicutes were significantly reduced at higher altitudes. But at the family level, Enterobacteriaceae dominated the bacterial community composition in leaves and remained sequences belonged to 6 families and unclassified family (Figure 3B). It was found that samples at higher altitudes have less species richness at the family level.

### Factors – bacteria abundance correlations

**FIGURE 4:**
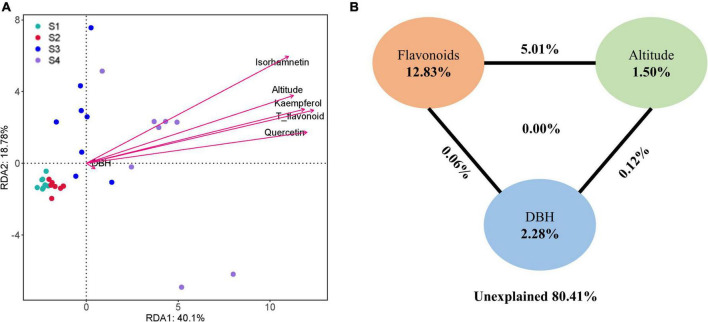
**(A)** Redundancy analysis (RDA) and **(B)** variance partitioning analysis (VPA) of the relationships between endophytic bacterial community and environmental variables in *Ginkgo biloba* leaves.

**FIGURE 5:**
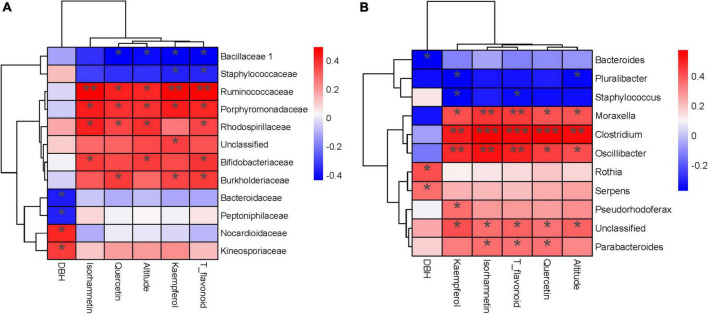
Heatmap **(A)** The correlation test between families and factors. **(B)** The correlation test between genera and factors. (***) *p* < 0.001, (**) *p* < 0.01 and (*) *p* < 0.05.

The Spearman correlation test and RDA analyzed the relationship between endophytic bacterial populations and factors. RDA results showed that the altitude, quercetin, kaempferol, isorhamnetin, and T_flavonoids significantly affected the endophytic bacterial community in ginkgo leaves, and the endophytic bacterial community was slightly affected by the age of Ginkgo (represented by DBH) (Figure 4A). VPA revealed that these factors constrained 19.59% of the total community distribution. The flavonoids individually constrained 12.83% of the community distribution, while the altitude and DBH constrained 1.50 and 2.28%, respectively (Figure 4B). Besides, flavonoids combined with altitude constrained 5.01% of the community distribution. The heatmap between factors and bacterial abundance was constructed by Spearman correlation values at both the family and genus levels. At the family level, we observed Ruminococcaceae and Porphyromonadaceae were positively correlated with three flavonols and T_flavonoid, while Rhodospirillaceae, Bifidobacteriaceae and Burkholderiaceae were positively correlated with T_flavonoids and one or two flavonols. There was a significant negative correlation between Bacillaceae and flavonoids (Figure 5A). It was found that endophytes of four genera were significantly positively correlated with flavonoids, including *Oscillibacter, Moraxella, Clostridium* and *Parabacteroides.* Only *Staphylococcus* showed a significant negative correlation with T_flavonoid and kaempferol (Figure 5B).

### Functions genes prediction by PICRUSt

**FIGURE 6:**
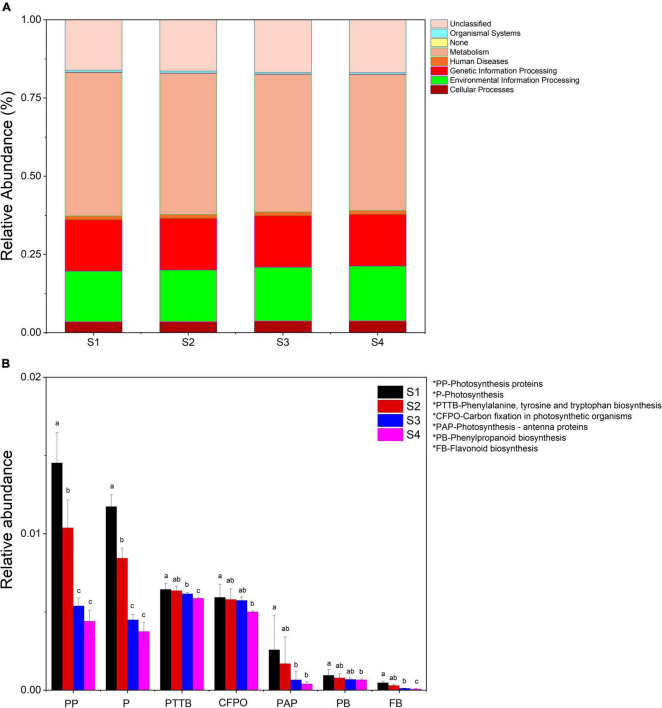
**(A)** Relative abundance of predicted function genes in different levels I samples. **(B)** Changes in the relative abundance of predicted genes in the flavonoid synthesis and photosynthesis pathways in all samples. Samples with different letters indicated significant differences between samples (*p* < 0.05).

Using PICRUSt (Phylogenetic Investigation of Communities by Reconstruction of Unobserved States) as a predictive exploratory tool, we found that seven orthology groups at the level I (KOs in KEGG (Kyoto Encyclopedia of Genes and Genomes)) were observed in the *Ginkgo* endophyte community. Relative abundance of metabolism, genetics information processing and cellular processes tended to be constant in the four samples. Almost half of the significant function genes were classified into multiple metabolism groups. Environmental information processing accounted for nearly 20% of all genes. The difference was the relative abundance of environmental information processing gradually increased from 16.2% in S1 to 17.5% in S4 (Figure 6A). Besides, there were more pronounced changes in function genes at level III. Photosynthetic pathways (including photosynthesis - antenna proteins, photosynthesis proteins, carbon fixation in photosynthetic organisms and photosynthesis) and flavonoids biosynthesis (consisting of phenylalanine, tyrosine and tryptophan biosynthesis, phenylpropanoid biosynthesis and flavonoid biosynthesis) gradually reduced (*p* < 0.05). The relative abundance of function genes of photosynthesis in S1 decreased by two-thirds compared with that in S1 (Figure 6B).

### The variation of the endophyte network along with altitude in ginkgo leaves

**FIGURE 7:**
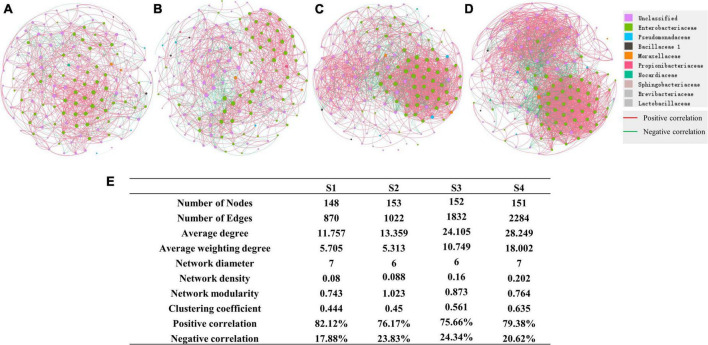
Network of different sites **(A)** Network of bacterial community at S1. **(B)** Network of S2. **(C)** Network of S3. **(D)** Network of S4. **(E)** Details of networks in different sites.

We further performed network analysis to assess the impact of altitude on ginkgo leaf endophyte. Our results showed that microbial interkingdom network patterns shifted clearly along with the increased altitude (Figure 7). Specifically, endophytic networks at lower altitudes had higher network connectivity (i.e., network degree) than networks at higher altitudes. Although there was a near number of nodes contributing to the network construction, the edges of networks gradually increased with the altitude increase. Compared with the network of S1, the number of edges increased by two times at S4. The network’s average degree and weighting degree increased from 11.757 and 5.705 to 28.249 and 18.002, respectively (Figure 7E).

## Discussion

### The increase of oxidation activity induced by light might be the key to increasing flavonoid content

In this study, total flavonoids increased significantly with altitude (Figures 1A,B). There was not much difference in temperature among the locations of the samples. Meanwhile, it implied that the excess light might be the main reason for the accumulation of flavonoids. Increasing light intensity within a certain range could enhance photosynthesis (Longstreth and Strain, 1977). Another study showed that the strengthening of light intensity could motivate photosynthesis and enhance the oxidative activity in leaves (Ghasemzadeh et al., 2010). As important antioxidants in plants, flavonoids were produced to resist these oxidative active substances like (O_2_^–^) (Pietta, 2000; Tattini et al., 2010). Thus, we speculated that the accumulation of flavonoids could be an important strategy for plants to protect against cellular damage. With the rise of elevation, the illumination was enhanced, which promoted photosynthesis and increased oxidation activity. *Ginkgo* trees produced more flavonoids to weaken oxidative damage. Our recent study observed that flavonoids and related genes increased with the altitude, attributing it to the enhanced illumination (Zou et al., 2019). The fact that the improvement of illumination intensity could promote the production of flavonoids and accelerate the accumulation of oxidative activity had also been reported in the previous study (Karimi et al., 2013). As an antioxidant, flavonoids protect the plant from light damage (Kumar et al., 2018). Besides, the light was proved to induce the oxidation and degradation of lipids (Sun et al., 2006; Wiswedel et al., 2009). All the above evidence showed that *Ginkgo biloba* increased flavonoid output to resist the accumulation of oxidative activity caused by the elevation and enhanced light.

### Flavonoids may regulate the structure of the endophytic community

Many secondary metabolites of plants can affect microbial communities, and flavonoids are no exception. With the increase of flavonoids, alpha diversity and observed_otus of the endophytic community decreased (Figure 2). Besides, we observed gram-negative bacteria accounted for the majority of total microorganisms, and the abundance of gram-negative bacteria increased from S1 to S4 (Figure 3). As the flavonoids increased, some gram-positive bacteria (including Firmicutes and Actinobacteria) decreased. We further analyzed the influencing factors of the microbial community in leaves by analyzing the correlation between the microbial community and factors such as flavonoid content and altitude (Figure 4). Flavonoids and altitude were proven to regulate the endophytic bacterial community in *Ginkgo biloba* leaves. Flavonoids are known to be antibacterial agents. Studies showed that the accumulation of flavonoids affected the symbiosis of endophytic bacteria and plants (Abdel-Lateif et al., 2012). Thus, flavonoids may be a key factor in the loss of diversity.

Furthermore, we found some microbiomes positively correlated with flavonoids, such as *Oscillibacter, Moraxella, Clostridium* and *Parabacteroides*. Microbes of *Clostridium* were proved to be flavonoid-degrading bacterium (Gupta et al., 2019). *Parabacteroides* were also found to increase with the addition of flavonoids in the gut microbiome (Zhang et al., 2022). In contrast, *Oscillibacter* and *Moraxella* decreased with the addition of flavonoids in the gut microbiome (Goulart et al., 2019; He et al., 2021; Cao et al., 2022). This difference might be because these microorganisms in Ginkgo leaves already live in an environment with high flavonoid content and have developed the resistance to flavonoids. Staphylococcus showed a significant negative correlation with T_flavonoid and kaempferol, the same as our previous study (Deng et al., 2021). Thus, we indicated that flavonoids could affect the community’s composition and structure in *Ginkgo* leaves.

Microorganisms facing stress need to develop some resistant strategies for survival. Our results showed that the network of endophytic bacteria in ginkgo leaves became more complex with the increasing altitude. Thus, it can be inferred that the adjustment of microbial interactions could be a strategy for microbes to resist enhancing environmental stresses, such as the increase of flavonoid content, altitude, and light intensity.

### Flavonoids regulated the physiological activities of endophytic microorganisms in leaves

In general, the environmental selection of microorganisms is mainly reflected in the collection of functions (Hammesfahr et al., 2011). The environmental information processing of endophytic bacteria in leaves significantly improved along with the increase of flavonoid content (Figure 5A), which also proved that the increase of altitude and flavonoids increased the environmental stress on endophytic bacteria in *Ginkgo biloba* leaves. *Ginkgo* leaves needed more environmental information processing genes to deal with the greater pressure caused by more flavonoids and more light with increased altitude. The analogical finding deemed environmental stress to be the momentous reason for enriching environmental information processing in early inquiry (Debroas et al., 2009). Furthermore, the abundance of genes on the photosynthetic pathway decreased with the flavonoids (Figure 5B). Microorganisms of Cyanobacteria were the dominating photosynthetic organisms in the endophytic communities. So the decrease of photosynthetic genes might attribute to the decreasing Cyanobacteria. We also observed genes related to flavonoid biosynthesis dropped with higher flavonoids. *In vitro*, flavonoid biosynthesis was inevitably inhibited by flavonoids’ accumulation by feedback inhibition (Yin et al., 2012). So we hypothesized that flavonoids could also inhibit the accumulation of microorganisms that could produce flavonoids in leaves. In conclusion, flavonoids played an essential role in coordinating endophytic community functions in *Ginkgo* leaves.

## Conclusion

In this study, the effects of altitude and flavonoids on the community of endophytic microorganisms in *Ginkgo biloba* were revealed by combining the analysis of plant metabolites and genomics of endophytic microorganisms. HPLC results expounded that the output of flavonoids in *Ginkgo* leaves improved with elevation. The enhancement of photosynthesis and accumulation of oxidative activity caused by altitude elevation might account for the increase of flavonoids. It was not difficult to find that flavonoids could regulate the overall function of the microbial community by selectively inhibiting or promoting certain microorganisms and function genes. The network of endophytes in *ginkgo* leaves also became more complex with increasing altitude and flavonoids. It may be the strategy of ginkgo endophytic bacteria to respond to environmental changes. These results would benefit the studies on the endophytic microbiome of plants and the cultivation of *Ginkgo biloba*. It can also help to clarify the interactions between the environment, plants and the plant microbiome. Nevertheless, some other unmonitored conditions might have been overlooked in terms of flavonoid synthesis and community structure. There must be further research on the effects of flavonoids and altitude on the endophytic microorganisms in *Ginkgo biloba*.

## Data availability statement

The data presented in this study are deposited in the NCBI repository, accession number: PRJNA852885.

## Author contributions

SF performed the experiments and wrote the manuscript. KZ helped with the sample collection. SZ assisted in performing some experiments. YD and YL revised the manuscript. All authors approved the submitted manuscript.

## Footnotes

1
http://mem.rcees.ac.cn:8080/

2
http://ieg.ou.edu/

3
https://www.bioincloud.tech
