# Supplementary material for: Flavonoids affect the endophytic bacterial community in Ginkgo biloba leaves with increasing altitude
Source: Front Plant Sci. 2022 Aug 11;13:982771. doi: 10.3389/fpls.2022.982771 (PMC9410704; doi:10.3389/fpls.2022.982771)
Supplement: Supplementary file 1 [file Data_Sheet_1.docx]

Supplementary Material

# Supplementary Tables

Table S1 Flavonoid content in Ginkgo Biloba leaves detected by HPLC (mg/kg).

| sample | | Quercetin | Kaempferol | Isorhamnetin | T_flavonoid |
| --- | --- | --- | --- | --- | --- |
| S1_1_1 | 234.27 | | 243.14 | 133.69 | 1549.43 |
| S1_1_2 | 239.10 | | 242.01 | 140.78 | 1575.51 |
| S1_2_1 | 247.54 | | 248.47 | 146.09 | 1626.44 |
| S1_2_2 | 249.24 | | 246.81 | 145.17 | 1624.10 |
| S1_3_1 | 255.49 | | 252.76 | 152.79 | 1673.73 |
| S1_3_2 | 258.26 | | 252.74 | 152.27 | 1679.38 |
| S1_4_1 | 257.66 | | 274.14 | 145.57 | 1718.37 |
| S1_4_2 | 255.96 | | 273.52 | 147.02 | 1715.94 |
| S2_1_1 | 273.09 | | 328.37 | 195.62 | 2019.89 |
| S2_1_2 | 272.26 | | 327.59 | 196.20 | 2017.12 |
| S2_2_1 | 281.34 | | 340.24 | 211.09 | 2108.90 |
| S2_2_2 | 281.26 | | 341.22 | 210.54 | 2109.97 |
| S2_3_1 | 283.22 | | 344.41 | 205.17 | 2110.47 |
| S2_3_2 | 281.79 | | 343.25 | 207.27 | 2108.85 |
| S2_4_1 | 289.82 | | 353.25 | 220.38 | 2186.72 |
| S2_4_2 | 295.69 | | 352.66 | 220.72 | 2200.71 |
| S3_1_1 | 360.40 | | 430.56 | 294.94 | 2746.18 |
| S3_1_2 | 361.76 | | 431.16 | 315.33 | 2799.92 |
| S3_2_1 | 361.88 | | 372.60 | 316.08 | 2647.41 |
| S3_2_2 | 362.90 | | 384.41 | 297.08 | 2635.75 |
| S3_3_1 | 374.08 | | 415.86 | 275.69 | 2695.72 |
| S3_3_2 | 372.34 | | 413.90 | 278.74 | 2693.47 |
| S3_4_1 | 374.90 | | 397.28 | 319.15 | 2752.58 |
| S3_4_2 | 374.93 | | 417.54 | 289.62 | 2735.57 |
| S4_1_1 | 930.00 | | 544.01 | 364.90 | 4642.61 |
| S4_1_2 | 881.84 | | 539.29 | 392.52 | 4575.29 |
| S4_2_1 | 893.34 | | 516.33 | 404.11 | 4571.21 |
| S4_2_2 | 898.15 | | 535.28 | 395.23 | 4612.09 |
| S4_3_1 | 894.70 | | 541.51 | 332.83 | 4470.74 |
| S4_3_2 | 900.52 | | 534.30 | 335.77 | 4473.36 |
| S4_4_1 | 904.34 | | 538.23 | 397.10 | 4639.88 |
| S4_4_2 | 903.83 | | 541.67 | 400.42 | 4655.62 |

Table S2. Linear regression equation of standards.

| Compounds | Retention time (min) | | | range(mg/L) | | Calibration curve | | R^2^ |
| --- | --- | --- | --- | --- | --- | --- | --- | --- |
| Quercetin | | 14.126-14.381 | 3.75-120 | | Y=25166X-67952 | | 0.9988 | |
| kaempferol | | 16.902-17.086 | 3.75-120 | | Y=31114X-52381 | | 0.9994 | |
| isorhamnetin | | 17.292-17.451 | 3.75-120 | | Y=26365X-25993 | | 0.9997 | |
